# Excess Mortality Attributable to COVID-19 Among Assisted Living Residents

**DOI:** 10.1093/geroni/igab046.221

**Published:** 2021-12-17

**Authors:** Kali Thomas, Wenhan Zhang, David Dosa, Paula Carder, Philip Sloane, Sheryl Zimmerman

**Affiliations:** 1 Brown University, Brown University/Providence, Rhode Island, United States; 2 Brown University, Providence, Rhode Island, United States; 3 OHSU-PSU School of Public Health, Portland, Oregon, United States; 4 UNC Medical School, Sheps Center, Chapel Hill, North Carolina, United States; 5 Cecil G. Sheps Center for Health Services Research, Chapel Hill, North Carolina, United States

## Abstract

This study examines the excess mortality attributable to COVID-19 among a national cohort of assisted living (AL) residents. To do this, we compare the weekly rate of all-cause mortality during 1/1/20-8/11/20 with the same weeks in 2019 and calculated adjusted incidence rate ratios (IRRs) and 95% confidence intervals (CIs). All-cause mortality rates, nationally, were 14% higher in 2020 compared with 2019 (mean, 2.309 vs. 2.020, respectively, per 1000 residents per week; adjusted IRR, 1.169; 95% CI 1.165-1.173). Among the 10 states with the highest community spread, the excess mortality attributable to COVID-19 was 24% higher, with 2.388 deaths per 1000 residents per week in 2020 during January-August vs 1.928 in 2019 (adjusted IRR, 1.241; 95% CI 1.233-1.250). These results suggest that AL residents suffered excess mortality due to COVID-19.

